# Empathy, Emotion Recognition, and Paranoia in the General Population

**DOI:** 10.3389/fpsyg.2022.804178

**Published:** 2022-02-24

**Authors:** Kendall Beals, Sarah H. Sperry, Julia M. Sheffield

**Affiliations:** ^1^Department of Psychiatry and Behavioral Sciences, Vanderbilt University Medical Center, Nashville, TN, United States; ^2^Michigan Medicine, University of Michigan, Ann Arbor, MI, United States

**Keywords:** paranoia, emotion recognition (ER), empathy, structural equation modeling (SEM), interpersonal reactivity index (IRI)

## Abstract

**Background:**

Paranoia is associated with a multitude of social cognitive deficits, observed in both clinical and subclinical populations. Empathy is significantly and broadly impaired in schizophrenia, yet its relationship with subclinical paranoia is poorly understood. Furthermore, deficits in emotion recognition – a very early component of empathic processing – are present in both clinical and subclinical paranoia. Deficits in emotion recognition may therefore underlie relationships between paranoia and empathic processing. The current investigation aims to add to the literature on social cognition and paranoia by: (1) characterizing the relationship between paranoia and empathy, and (2) testing whether there is an indirect effect of emotion recognition on the relationship between empathy and paranoia.

**Methods:**

Paranoia, empathy, and emotion recognition were assessed in a non-clinical sample of adults (*n* = 226) from the Nathan Kline Institute-Rockland (NKI-Rockland) dataset. Paranoia was measured using the Peters Delusions Inventory-21 (PDI-21). Empathy was measured using the Interpersonal Reactivity Index (IRI), a self-report instrument designed to assess empathy using four subscales: Personal Distress, Empathic Concern, Perspective Taking, and Fantasy. Emotion recognition was assessed using the Penn Emotion Recognition Test (ER-40). Structural equation modeling (SEM) was used to estimate relationships between paranoia, the four measures of empathy and emotion recognition.

**Results:**

Paranoia was associated with the Fantasy subscale of the IRI, such that higher Fantasy was associated with more severe paranoia (*p* < 0.001). No other empathy subscales were associated with paranoia. Fantasy was also associated with the emotion recognition of fear, such that higher Fantasy was correlated with better recognition of fear (*p* = 0.008). Paranoia and emotion recognition were not significantly associated. The Empathic Concern subscale was negatively associated with emotion recognition, with higher empathic concern related to worse overall emotion recognition (*p* = 0.002). All indirect paths through emotion recognition were non-significant.

**Discussion:**

These results suggest that imaginative perspective-taking contributes to paranoia in the general population. These data do not, however, point to robust global relationships between empathy and paranoia or to emotion recognition as an underlying mechanism. Deficits in empathy and emotion recognition observed in schizophrenia may be associated with the broader pathology of schizophrenia, and therefore not detectable with subclinical populations.

## Introduction

Paranoia is one of the most common psychotic experiences, occurring in over 70% of individuals presenting with their first episode of psychosis (Coid et al., 2013) and 10–15% of the general population (Freeman and Freeman, 2008). Models of paranoia suggest that social cognitive abnormalities contribute to paranoid thinking by creating an information-processing bias (Locascio and Snyder, 1975; Freeman et al., 2002), leading to misinterpretation of others’ emotions and intentions, fueling mistrust. Social cognition is comprised of multiple domains including emotion processing, social perception, theory of mind, attributional bias, and empathy (Decety and Svetlova, 2012; Pinkham, 2014). Evidence suggests that theory of mind, attributional bias, emotion processing and social perception are all associated with paranoid thinking (McKay et al., 2005; Combs et al., 2006), and these relationships are observed across clinical and non-clinical populations (Martin and Penn, 2001; Freeman et al., 2002; Combs et al., 2013). Given that both paranoid thinking and social cognitive deficits contribute to functional impairment (Minkowski, 1953; Shamay-Tsoory et al., 2007; Sparks et al., 2010; Pinkham et al., 2016; Bonfils et al., 2017), fully elucidating how different facets of social cognition relate to paranoia is critical for advancing models of and ultimately treatments for paranoia.

In schizophrenia, social cognitive deficits have been largely described in relationship to negative symptoms and deficits in social functioning (Green et al., 2019), with evidence that social cognition and negative symptoms predict social competence and social outcomes (Kalin et al., 2015) and are related to social cognitive processes, including empathy (Wang et al., 2021). Yet, as noted, social cognition is also relevant for “positive” psychotic experiences (Bliksted et al., 2017; Peyroux et al., 2019), including paranoid thinking (Green and Leitman, 2007). One robust relationship between paranoia and social cognition is via misinterpretation of social cues (Trotta et al., 2021). Individuals with heightened paranoia show evidence of misinterpreting ambiguous information more negatively (Bentall et al., 2009; Savulich et al., 2015), which may be shaped by negative prior beliefs about the intentions of others (Wellstein et al., 2020). Importantly, recent meta-analysis has shown that the relationship between paranoia and misinterpretation of ambiguous cues (including emotional cues) is present in both clinical and non-clinical populations, with more severe paranoia being related to worse interpretation bias (Trotta et al., 2021). This suggests that these experiences exist on a continuum across clinical and non-clinical populations. This evidence of a continuum of social cognition and paranoia, in addition to the impact of negative symptoms on social cognition, highlight the utility of examining relationships between social cognition and paranoia in a general population sample that is less impacted by co-occurring experiences of schizophrenia (e.g., negative symptoms and cognitive impairment).

Despite prior relationships between interpretation bias and paranoia, as well as other aspects of social cognition (e.g., theory of mind and social perception), there is a dearth of research examining associations between paranoia and empathy (Buck et al., 2017; Lee, 2017; Herms et al., 2022). Empathy may be a particularly important aspect of social cognition for understanding paranoia, because successful social interactions require the capacity for interpreting emotional states, beliefs, and motivations of others (Rochat and Striano, 1999; Decety and Meyer, 2008). Empathy is a multifaceted construct that involves both cognitive and affective components (Baron-Cohen and Wheelwright, 2004; Blair, 2005). Cognitive empathy involves a set of reflective processes that include perspective taking and distinguishing another’s feelings from one’s own, whereas affective empathy is a more automatic set of processes through which perceived social cues trigger an emotional response in oneself that is shared with an observed person (Michaels et al., 2014). In schizophrenia, both types of empathy are impaired (Bonfils et al., 2017) while those in the ultra-high risk phase of psychosis show impaired cognitive empathy (Jan Kuis et al., 2021). Studies have also found that heightened engagement with imaginative, perceptual, and ideational resources predicts delusional ideation in the general population (Tellegen and Atkinson, 1974; Humpston et al., 2016; Murphy et al., 2018). Whether distinct facets of empathy are differentially associated with paranoia has not been previously investigated.

Both cognitive and affective empathy depend, at least in part, on the ability to accurately recognize emotional expression. The ability to recognize basic facial expressions develops very early in life (Field et al., 1982), is universal across cultures (Ekman and Friesen, 1971) and is acquired in closely related animal species (Darwin, 1872). Emotion recognition is an early facet of the empathetic process and reflects a more intrinsic, biological aspect of social cognition (Besel and Yuille, 2010). Research investigating the relationship between empathy and emotion recognition has found that people with high levels of empathy are more sensitive to subliminally presented emotional face stimuli (Martin et al., 1996) and rated angry faces as expressing more anger and happy faces as being happier than people with low empathy (Dimberg et al., 2011). In individuals with polygenic risk for schizophrenia, emotion recognition deficits are evident by mid childhood and are related to severity of psychotic experiences (Germine et al., 2016). Research has also demonstrated that paranoid patients show worse emotion recognition ability than non-paranoid patients (An et al., 2006; Russell et al., 2007; Williams et al., 2007), possibly due to a tendency for paranoid patients to inaccurately recognize neutral facial expressions as angry (Pinkham et al., 2011). Some research points to empathy being a factor in one’s ability to accurately recognize facial expressions (Besel and Yuille, 2010; Wai and Tiliopoulos, 2012). Although emotion recognition ability contributes to empathy, and is impaired in paranoid patients, the unique and shared contribution of these social cognitive domains to paranoia has not been studied directly.

The current study aims to examine the relationship between paranoia, empathy, and emotion recognition in a large sample of individuals from the general population. Given previous findings that impaired social cognitive ability is related to worse paranoia in both clinical and non-clinical samples, and that emotion recognition is important for empathic ability, we hypothesized the following: (1) that greater deficits in empathic abilities and emotion identification would be related to more severe paranoid thinking, and (2) that there would be a significant indirect effect of emotion identification on the relationship between empathy and paranoia. As prior work on empathy and paranoia is sparse, we did not have strong predictions about the type of empathy that would be most related to paranoia, and hoped instead to address this knowledge gap.

## Materials and Methods

### Participants

Participants were drawn from the Nathan Kline Institute-Rockland study (NKI-Rockland) a large (>1,000 individuals) community-ascertained sample representative of the broader United States population (age 6–85) based on the 2010 census (Nooner et al., 2012). From the total cohort, 226 adult participants (age 18–65) were identified who completed measures of paranoid thinking, empathy, and emotion recognition (described below) within a 5-day period (*M*_age_ = 38.64, SD_age_ = 15.91; 58.4% self-identified as female, 41.6% male; 64.6% White, 20.8% Black/African American, 14.6% Asian/Native Hawaiian/American Indian/Other Race).

### Procedures

The NKI Rockland Sample was collected in a multi-phase National Institute of Mental Health (NIMH) funded study. Phase one collected psychiatric, behavioral, and cognitive data from 250 convenience sampled individuals from 4 to 89 years old. The second phase aimed to collect data from 1,000 participants from 6 to 85 years old who reflect the demographics of the 2009 United States census (Nooner et al., 2012). For each study visit, participants fill out self-report questionnaires (including the PDI-21 and IRI) at home within 28 days of the in-person study day. The baseline visit included 2 days of study related tasks including neuropsychological testing, neuroimaging, and behavioral tasks. A full overview of participant protocols can be found here: http://fcon_1000.projects.nitrc.org/indi/enhanced/ALGFullEndUserProtocol.pdf. The first and second phase were approved by the Institutional Review Board (Nathan Kline Institute Phase I #226781 and Phase II #239708; Montclair State University Phase I #000983A and Phase II #000983B) and participants were paid up to $200 for the baseline 2-day study or $250 if that baseline included an MRI.

### Measures

Mean, standard deviation, range, and Cronbach’s alpha are presented in Table 1.

**TABLE 1:** IRI, PDI, and ER-40 descriptive statistics.

	MEAN (SD)	RANGE	CRONBACH’S ALPHA
PARANOIA	9.51 (9.88)	0–57	0.88
FANTASY	14.27 (6.15)	0–28	0.79
EMPATHIC CONCERN	21.42 (4.3)	12–28	0.70
PERSONAL DISTRESS	11.87 (5.12)	2–29	0.76
PERSPECTIVE TAKING	16.68 (3.66)	5–24	0.66
ER TOTAL	35.17 (2.75)	24–40	
ER FEAR	6.85 (1.32)	2–8	
ER SADNESS	6.67 (1.17)	1–8	
ER ANGER	6.62 (1.12)	3–8	
ER HAPPY	7.96 (0.2)	7–8	
ER NO EMOTION	7.06 (1.26)	0–8	

#### Peters Delusions Inventory-21 Item

The Peters Delusions Inventory (PDI) is a self-report measure that assesses delusional thinking in the general population and has shown acceptable reliability and validity (Peters et al., 2004). The PDI measures a variety of delusional beliefs by asking participants whether or not they relate to different statements (e.g., “Do you ever feel as if you are being persecuted in some way?”, “Do you ever feel as if there is a conspiracy against you?”). If they state “yes” then participants are asked to score the amount of distress, preoccupation, and conviction they experience on a scale of one to five. Prior factor analysis of the PDI-21 has identified four items that are associated with paranoid thinking (Verdoux et al., 1998; López-Ilundain et al., 2006). Scores for each of the four paranoia items were summed to determine a measure of paranoid thinking as previously reported (Preti et al., 2007; Sheffield et al., 2021). Total scores ranged from 0 to 64.

#### Interpersonal Reactivity Index

The Interpersonal Reactivity Index (IRI) is a well-validated 28-item self-report scale measuring empathy (Davis, 1980, 1983). The IRI is comprised of four scales: Fantasy (ability to put oneself into fictional situations and take the perspective of fictitious characters), Empathic Concern (“other-oriented” feelings of sympathy and concern for others), Perspective Taking (ability to adopt the psychological viewpoint of others), and Personal Distress (“self-oriented” feelings of personal distress in interpersonal settings). Each subscale was calculated separately, as recommended (Davis, 1980, 1983; Chrysikou and Thompson, 2016), by summing the seven items in each of the four subscales (Shamay-Tsoory et al., 2007; Bonfils et al., 2017). The Fantasy and Perspective Taking scales reflect cognitive empathy and the Empathic Concern and Personal Distress scales reflect affective empathy.

Example items for Fantasy include “I really get involved with the feelings of the characters in a novel”, Empathic Concern includes “I often have tender, concerned feelings for people less fortunate than me,” Perspective Taking includes “I try to look at everybody’s side of a disagreement before I make a decision” and Personal Distress includes “I sometimes feel helpless when I am in the middle of a very emotional situation.”

Davis (1983) explained how the IRI has a hierarchical structure, with each factor mirroring an aspect of the general empathy construct. More recent studies have supported the four-factor model (Cliffordson, 2002; Hawk et al., 2013). Separating the IRI into Cognitive and Affective empathy is known to be unsupported by psychometric analyses (Chrysikou and Thompson, 2016). Therefore, the four subscales were analyzed separately, to examine unique relationships with paranoia and emotion recognition.

#### Penn Emotion Recognition Test (ER-40)

The Penn Emotion Recognition Test was conducted as part of the Penn Computerized Neurocognitive Battery (Moore et al., 2015). The PEIT measures participants’ ability to recognize five emotions (Happy, Sad, Anger, Fear, and No Emotion) and has good test-retest reliability (Weiss et al., 2007). Emotional faces are presented on individuals of both genders and multiple races. A total of 40 photos are presented on a computer screen where participants recognize the type of emotion shown in a forced-choice format. Correct responses are scored as 1 and incorrect as 0 for a maximum score as 40, where a score of 40 indicates better overall facial emotion recognition. Subscales were also calculated for each of the five expressions: Happy recognition, Sad recognition, Anger recognition, Fear recognition, and No Emotion recognition (Gur et al., 2001a,b; Moore et al., 2015).

### Statistical Analysis

Relationships between paranoia, emotion recognition, and empathy, were first examined in zero-order correlations using SPSS v.25.0 (IBM Corp. Released, 2017). Next, *a priori* hypotheses were tested using structural equation modeling (SEM), conducted in R (lavaan R 4.0.2 package; Rosseel, 2012). In our *a priori* SEM model, each IRI subscale (exogenous variable) predicted paranoia (endogenous). Paths were also specified from each IRI subscale to overall emotion recognition ability and from emotion recognition ability to paranoia. To test our hypothesis that there would be a relationship between empathy and paranoia through emotion recognition, we estimated an indirect path from empathy (IRI subscales) to emotion recognition ability to paranoia. Our primary model used overall emotion recognition ability. Follow-up sensitivity analyses were conducted to examine whether specific types of emotion recognition (e.g., fear) were related to empathy and paranoia. All analyses included age, sex, and race as covariates. Standard errors were calculated based on 1000 bootstrapped samples. We evaluated model fit using CFI, RMSEA, and SRMR based on Hu and Bentler (1999) criteria.

## Results

In this general population sample, mean paranoia was a 9.51 (SD = 9.88; max. 60). For each empathy subscale the maximum score is 35, and participants had a mean score of 14.27 (SD = 6.15) for Fantasy, 21.42 (SD = 4.3) for Empathic Concern, 11.87 (SD = 5.12) for Personal Distress, and 16.68 (SD = 3.66) for Perspective Taking. Emotion recognition has a maximum score of 40 and participants in this sample had a mean score of 35.17 (SD = 2.75).

### Zero-Order Correlations

Correlations between all variables are presented in Table 2. The Fantasy subscale was significantly positively correlated with paranoia (*p* < 0.001) and fear recognition (*p* < 0.001). Emotion recognition was significantly negatively correlated with Empathic Concern (*p* = 0.037). Paranoia and emotion recognition were not correlated with any other measure.

**TABLE 2:** Zero-order correlations.

	Perspective taking	Fantasy	Empathic concern	Personal distress	Paranoia	ER total	ER anger	ER fear	ER happy	ER no emotion	ER sad
Perspective taking											
Fantasy	0.20										
Empathic concern	0.42**	0.33**									
Personal distress	–0.07	0.29**	0.14*								
Paranoia	–0.04	0.24**	0.05	0.09							
ER total	0.01	0.09	−0.14*	0.01	0.02						
ER anger	0.02	–0.04	−0.15*	0.01	–0.03	0.57**					
ER fear	–0.07	0.24**	–0.04	0.11	0.11	0.59**	0.12				
ER happy	0.03	0.00	–0.04	–0.07	0.07	0.17*	0.16*	0.08			
ER no emotion	0.01	0.00	–0.07	–0.09	0.04	0.48**	–0.02	0.03	–0.10		
ER sad	0.07	–0.02	–0.06	0.00	–0.10	0.60**	0.25**	0.09	0.10	0.06	

ER, emotion recognition. **Correlation is significant at the 0.01 level (two-tailed). *Correlation is significant at the 0.05 level (two-tailed).

### Structural Equation Model

**FIGURE 1:**
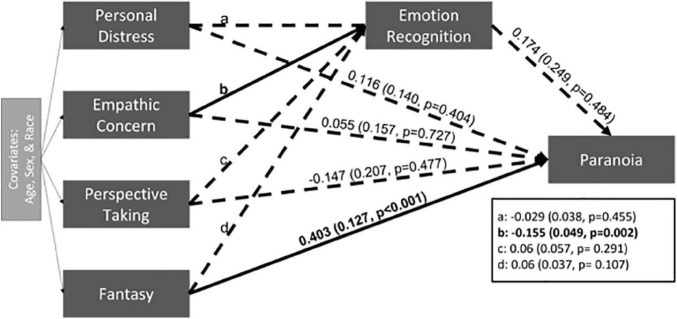
The estimate and standard error are presented for each path. Significant paths are indicated with solid black lines and bolded coefficients. Non-significant pathways are indicated with dashed lines.

**FIGURE 2:**
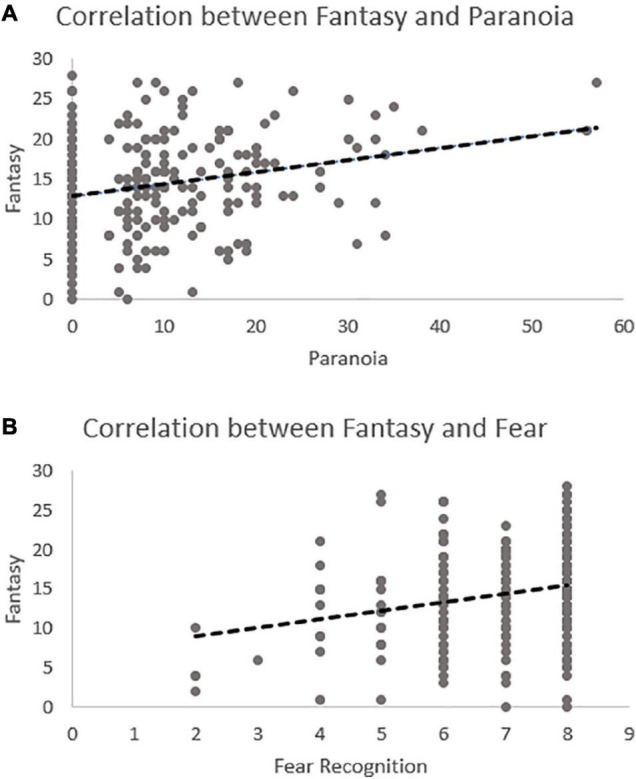
Scatterplots demonstrating the significant relationship in the model between Fantasy and paranoia **(A)** and Fantasy and fear recognition **(B)**.

The main hypothesized model (Figure 1) had poor fit (CFI = 0.46, RMSEA = 0.25, SRMR = 0.08) so results should be interpreted with caution. Fantasy was significantly positively associated with paranoia (*p* < 0.001) (Figure 2A) and Empathic Concern was significantly negatively associated with overall emotion recognition ability (*p* = 0.002) (Figure 2B). Contrary to expectation, there was not a significant indirect path between empathy subscales, emotion recognition, and paranoia.

### Sensitivity Analyses

**FIGURE 3:**
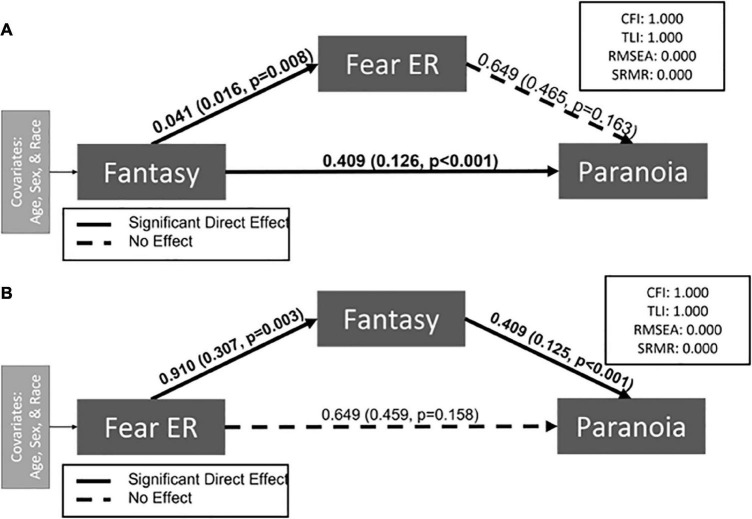
Exploratory path models demonstrate associations between Fantasy, fear recognition, and paranoia. Indirect paths are designated either through fear recognition **(A)** or Fantasy **(B)**. Neither model demonstrated a significant indirect effect. The estimate and standard error are presented for each path. Significant paths are indicated with solid black lines and bolded coefficients. Non-significant pathways are indicated with dashed lines.

Given the relatively poor model fit of the *a priori* theorized model, we ran several *post hoc* sensitivity analyses to determine whether modeling relationships with specific emotions would better fit our data (Supplementary Figures 1–4). First, we examined the paths between Fantasy and paranoia through emotion recognition for each emotion separately (e.g., fear). When considering ability to recognize specific emotions, we found that Fantasy was positively associated with fear emotion recognition (*p* = 0.008) and paranoia (*p* < 0.001), but no significant indirect path (Figure 3A) emerged. Of note, this model was oversaturated (CFI = 1.00, RMSEA = 0.00, SRMR = 0.00) indicating that it did not fit the observed data. Next, we tested an exploratory model in which we alternated the order of the endogenous and exogenous variables – fear recognition (exogenous) predicting paranoia (endogenous) through Fantasy. This model was also oversaturated (CFI = 1.0, RMSEA = 0.00, SRMR = 0.00) and showed similar results: fear emotion recognition was positively associated with Fantasy (*p* = 0.003) and Fantasy was positively associated with paranoia (*p* < 0.001) (Figure 3B) with no significant indirect path. Please see Table 3 for all SEM parameters.

**TABLE 3:** Test statistic.

	Test statistic	DF	Chi-square	CFI	TLI	RMSEA	90% CI low	90% CI upper	*P* RMSEA < = 0.05	SRMR
ER total	91.981	6	0.000	0.459	−1.975*	0.252	0.208	0.298	0.000	0.083
ER happy	91.981	6	0.000	0.394	−2.335*	0.252	0.208	0.298	0.000	0.081
ER no emotion	91.981	6	0.000	0.401	−2.293*	0.252	0.208	0.298	0.000	0.081
Anger	91.981	6	0.000	0.401	−2.292*	0.252	0.208	0.298	0.000	0.083
Fear	91.981	6	0.000	0.468	−1.925*	0.252	0.208	0.298	0.000	0.082
Sad	91.981	6	0.000	0.421	−2.184*	0.252	0.208	0.298	0.000	0.082
Fantasy-Fear-Paranoia	0.000	0	N/A	1.000	1.000	0.000	0.000	0.000	N/A	0.000
Fear-Fantasy-Paranoia	0.000	0	N/A	1.000	1.000	0.000	0.000	0.000	N/A	0.000

*Typically, TLI values range between 0 and 1. However, in models with smaller sample sizes and low degrees of freedom, TLI can have negative values. These can be rounded to 0. Anderson and Gerbing (1984) indicates that TLI is an inferior fit index for SEM models with small sample sizes.

## Discussion

This study investigated the relationship between empathy, emotion recognition, and paranoia in subclinical populations, considering different types of empathy. Our findings suggest that increased scores on the Fantasy subscale of the IRI are associated with increased paranoia in the general population, but that this relationship is independent of one’s overall ability to recognize emotional expressions. In addition, increased scores on Empathic Concern were related to decreased emotion recognition. Interestingly, higher Fantasy scores were significantly associated with both increased paranoia and better fear recognition, and no other facet of empathy showed this effect. Taken together, specific facets of cognitive empathy (Fantasy) are related to paranoia and fear recognition in the general population, but the association between empathy and paranoia does not appear to depend on ability to detect fearful faces.

### Fantasy and Paranoia

In this general population dataset, empathic fantasy was positively associated with paranoia, suggesting that individuals who experience more paranoia report a tendency toward empathically relating to fictional characters. Previous studies in schizophrenia that have investigated empathy using the IRI have reported varying levels of disruption in empathic fantasy (Rankin et al., 2005; Sucksmith et al., 2013; Bonfils et al., 2017), with some research demonstrating deficits (Fujiwara et al., 2008; Hooker et al., 2011) while others have shown greater or similar Fantasy scores in schizophrenia as compared to healthy controls (Sparks et al., 2010; McCormick et al., 2012; Matsumoto et al., 2015). Meta-analysis reported slightly reduced Fantasy scores in schizophrenia, with a small effect size (Hedge’s *g* = 0.19).

Although the IRI has been thoroughly investigated in patients with schizophrenia, there are fewer studies looking at the IRI and sub-clinical paranoia. In one study, Buck et al. (2017) found that distorted mind perception (perceiving that someone/something possesses a thinking, feeling mind) partially explained the association between paranoia and the perspective taking and empathic concern subscales but found no direct effects. In another study, Lee (2017) found that patients with paranoid personality disorder had diminished perspective taking and increased personal distress, but no differences in Fantasy. Interestingly, although Buck et al. (2017) did not specifically investigate the Fantasy subscale, they do report that paranoia is positively associated with a tendency to perceive mind in dead people, trees, robots and Superman. Mind perception of a character like Superman reflects the person’s sense that the fictional character has memories, agency, and ability to feel fear, pleasure and hunger. These findings in a larger sample may bear on our data, suggesting that those with greater tendency toward paranoia experience more empathy toward fictional characters.

Also relevant to our findings is a previous report of a significant relationship between Fantasy scores and delusion severity in individuals with schizophrenia (Sparks et al., 2010). Although Fantasy scores did not differ between patients and controls in this prior analysis, greater Fantasy was strongly related to more severe delusions. In addition, in a study looking at empathy and theory of mind in first-degree relatives of individuals with schizophrenia (i.e., those at elevated genetic risk), it was only the Fantasy subscale of the IRI (not theory of mind or other measures of empathy) that was associated with a history of subclinical delusional ideation, such that greater delusional ideation was related to greater Fantasy scores (Montag et al., 2012).

Our findings therefore add to a growing literature that connects delusional ideation and the Fantasy subscale of the IRI. The relationship between these two measures is somewhat confounding, in part because the Fantasy subscale is, itself, difficult to interpret (Nomura and Akai, 2012). The Fantasy scale measures the individual’s tendency to use their imagination in order to take the perspective of a fictional character. Unlike the other IRI subscales, Fantasy is stable across adolescence, suggesting it may function slightly differently than other facets of empathy, which change over time (Davis and Franzoi, 1991). In factor analysis, Fantasy loads with Perspective Taking on a factor of cognitive empathy (Shamay-Tsoory et al., 2007), indicating it may reflect more advanced capabilities similar to theory of mind. In fact, scores on the Fantasy scale have been previously related to verbal measures and intellectual ability (Mayer and Geher, 1996; Montag et al., 2012). Paranoia is a facet of delusional ideation focused on a belief that one is under threat from others (Freeman, 2016). Delusions, including paranoia, are a self-conscious experience that are thought to be born from an unstable boundary between the self, the world, and others (Sass and Parnas, 2003). While speculative, relationships between Fantasy and paranoia may be an unexpected glimpse into this boundary disturbance that can occur in individuals on the delusion spectrum (Feyaerts et al., 2021). Its relationship with paranoia could also be a byproduct of the safety that fictional characters can provide. More paranoid individuals may feel more connected with fictional characters, because they cannot cause them imminent harm and may themselves be persecuted.

### Emotion Recognition

Another contribution of the current study is our investigation into role that emotion recognition plays with empathy and paranoia. Both emotion recognition and empathy are critical for healthy social interactions, and impaired social functioning contributes to paranoia (Fiske and Taylor, 2013; Chen, 2014; Wickham et al., 2014). Emotion recognition is a more “basic” ability that supports interaction with the environment, underlying the more complex process of experiencing empathy (Duesenberg et al., 2016). Due to empathy’s dependence on recognizing facial emotion, we expected to find an association between the IRI subscales and emotion recognition. After *post hoc* analyses, the only subscales we found to be associated with emotion recognition was the Fantasy subscale and the Empathic Concern subscale. Previous studies linking empathy and emotion recognition have found that emotion recognition capabilities correlate with higher dispositional empathy (Davis and Kraus, 1997; Hall et al., 2009). Soto et al. (1998) found that people who are empathically accurate (better at rating how a stranger feels from moment to moment) are better at identifying positive and negative emotions. Papers that have looked specifically at the IRI and ER-40 measures generally found increased emotion recognition in relationship to higher empathy (Martin et al., 1996; Gery et al., 2009).

In our study, emotion recognition was associated with both Fantasy and Empathic Concern, however, there was not an indirect relationship between these facets of empathy and paranoia through emotion recognition. Interestingly, Fantasy was specifically associated with recognition of the emotion of fear, such that better fear recognition was related to greater empathic fantasy. Expressions of fear are distress cues that can drive feelings of empathy (Panksepp and Panksepp, 2013), and better fear recognition is related to more pro-social behavior (Marsh et al., 2007). Relationships with Fantasy and fear recognition are novel, as prior work linking fear recognition and empathy have looked only at Empathic Concern (Besel and Yuille, 2010). Unlike our findings, however, which revealed a negative relationship, prior work has demonstrated a positive relationship between Empathic Concern on the IRI and emotion recognition ability (Jiang et al., 2014). This finding was strongest for emotion recognition after a brief exposure (50 ms), which is much shorter than the current task parameters. This implies that sensitivity to the relationship between emotion recognition and Empathic Concern may depend on quick, automatic processing. Overall, the findings between empathy and emotion recognition in the current study are intriguing but require replication and further investigation.

The lack of a significant association between emotion recognition and paranoia is also somewhat surprising. Past research completed in the general population has shown that those with higher subclinical paranoia had lower overall emotion recognition ability (Combs and Penn, 2004; Klein et al., 2018), and misidentified neutral expressions for anger (Pinkham et al., 2011), although a recent study found that positive schizotypy was not associated with emotion recognition (Dawes et al., 2021). This relationship has also been studied extensively in patients with schizophrenia. In a study comparing schizophrenia participants and subclinical populations with varying levels of paranoia, schizophrenia participants had worse emotion recognition ability than those with low or moderate subclinical paranoia, but similar emotion recognition as subclinical participants with high paranoia (Combs et al., 2006). Lack of replication in the current sample may be due to our use of the PDI-21 to measure paranoia. Prior studies have used the Paranoia Scale – a 20-item self-report focused entirely on paranoia (Combs and Penn, 2004; Klein et al., 2018), allowing for more power to detect relationships within a subclinical population.

### Strengths and Weaknesses

This investigation approached the relationship between empathy, emotion recognition and paranoia through a sophisticated statistic method that allowed us to test the relative contribution of different types of empathy on paranoia, as well as model our hypothesized pathways. However, this study was limited by relatively low power for a structural equation model, which may have affected the specificity and fit of each model (Anderson and Gerbing, 1984). Another limitation to this study is the nature of the Peters Delusion Inventory and Interpersonal Reactivity Index. Self-report questionnaires are often influenced by response biases like social desirability. As the Peters Delusion Inventory asks questions about thoughts that may be stigmatizing (e.g., Do you ever feel as if there is a conspiracy against you? Do you ever think people can communicate telepathically?), a participant may report lower levels of distress, preoccupation, and conviction. Furthermore, the IRI may be best interpreted as a participant’s thoughts about their empathic abilities and not their actual abilities. Self-reported cognitive empathy abilities have been found to be unrelated to actual empathic accuracy during a brief interaction with another person (Ickes et al., 2000; Zaki and Ochsner, 2009). A more interactive or dynamic task may be necessary to tap into the social cognitive domain of empathy (Haut et al., 2019). Finally, Batson et al. (2002) has questioned whether empathy can be measured validly by self-reports and the internal consistency of the fantasy subscale as a measure of empathy has been previously questioned (Baldner and McGinley, 2014) and therefore should be interpreted carefully. One main note is that the fantasy subscale, unlike the other three subscales, was created from two sources [Stotland’s (1969) Fantasy-Empathy scale and Davis (1983)]. Baron-Cohen and Wheelwright (2004) proposed that fantasy was not characterized by affective-cognitive dimensions of empathy and suggested that the scale assesses imagination, not empathy itself (Lawrence et al., 2004). In the schizophrenia literature, results of differences in fantasy are mixed (Horan et al., 2015; Bonfils et al., 2017). Therefore, while we are intrigued by the fairly consistent relationships between fantasy and delusional ideation across the literature, we acknowledge that its interpretation as a facet of empathy requires further validation.

### Future Directions

This study was one of the first to identify relationships between a cognitive facet of empathy and paranoia, as well as fear recognition. The role of fear recognition in the relationship between Fantasy and paranoia should be explored further. If replicated, future studies should examine the directionality of these relationships, for instance testing whether increased Fantasy is present prior to paranoia onset. This would require longitudinal or temporal data that could truly capture directionality. This study provides future support for considering symptoms as a continuum and highlights the importance of studying the social-cognitive aspects of experiences like paranoia.

## Data Availability Statement

Publicly available datasets were analyzed in this study. This data can be found here: http://fcon_1000.projects.nitrc.org/indi/enhanced/access.html.

## Ethics Statement

Institutional Review Board Approval was obtained for this project at the Nathan Kline Institute (Phase I #226781 and Phase II #239708) and Montclair State University (Phase I #000983A and Phase II #000983B). The patients/participants provided their written informed consent to participate in this study.

## Author Contributions

KB, SS, and JS conceptualized the study. SS conducted initial data analysis, and including suggestion of statistical approaches and provided feedback for the manuscript. KB conducted *post hoc* analyses and developed all figures and tables. KB and JS drafted the initial manuscript. All authors contributed to the article and approved the submitted version.

## Conflict of Interest

The authors declare that the research was conducted in the absence of any commercial or financial relationships that could be construed as a potential conflict of interest.

## Publisher’s Note

All claims expressed in this article are solely those of the authors and do not necessarily represent those of their affiliated organizations, or those of the publisher, the editors and the reviewers. Any product that may be evaluated in this article, or claim that may be made by its manufacturer, is not guaranteed or endorsed by the publisher.
