# Supplementary material for: Empathy, Emotion Recognition, and Paranoia in the General Population
Source: Front Psychol. 2022 Feb 24;13:804178. doi: 10.3389/fpsyg.2022.804178 (PMC8908382; doi:10.3389/fpsyg.2022.804178)
Supplement: Supplementary file 1 [file Data_Sheet_1.docx]

**Supplement**

**Figure S1: Path model with accuracy for no emotion recognition**


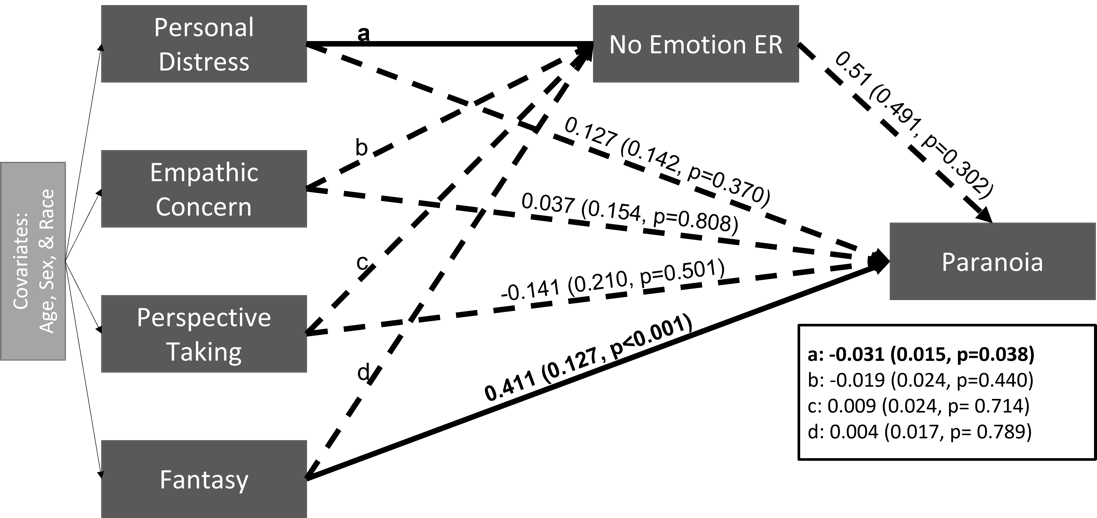


The estimate and standard error are presented for each path. Significant paths are indicated with solid black lines and bolded coefficients. Non-significant pathways are indicated with dashed lines.

**Figure S2: Path model with accuracy for happy emotion recognition**


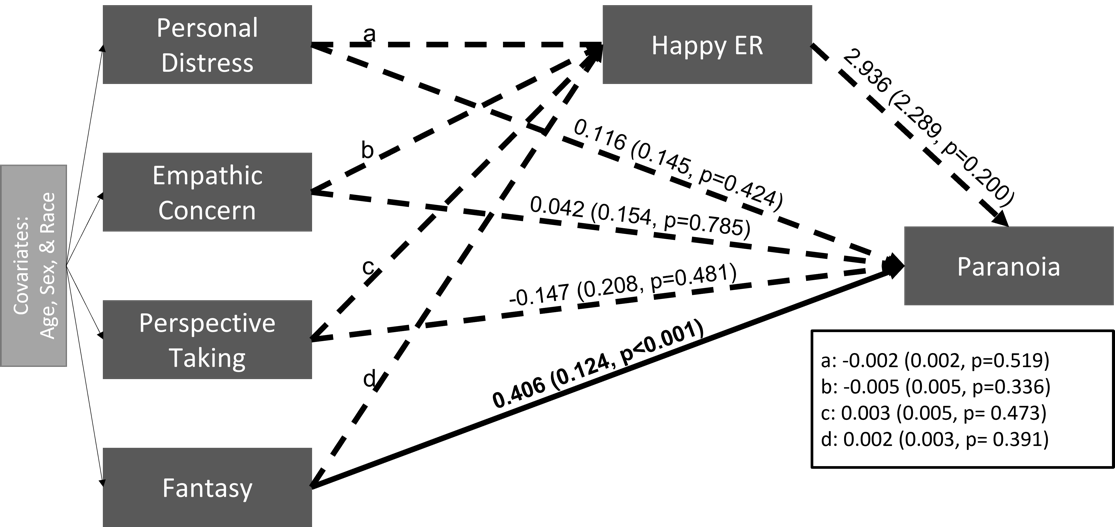


The estimate and standard error are presented for each path. Significant paths are indicated with solid black lines and bolded coefficients. Non-significant pathways are indicated with dashed lines.

**Figure S3: Path model with accuracy for sad emotion recognition**


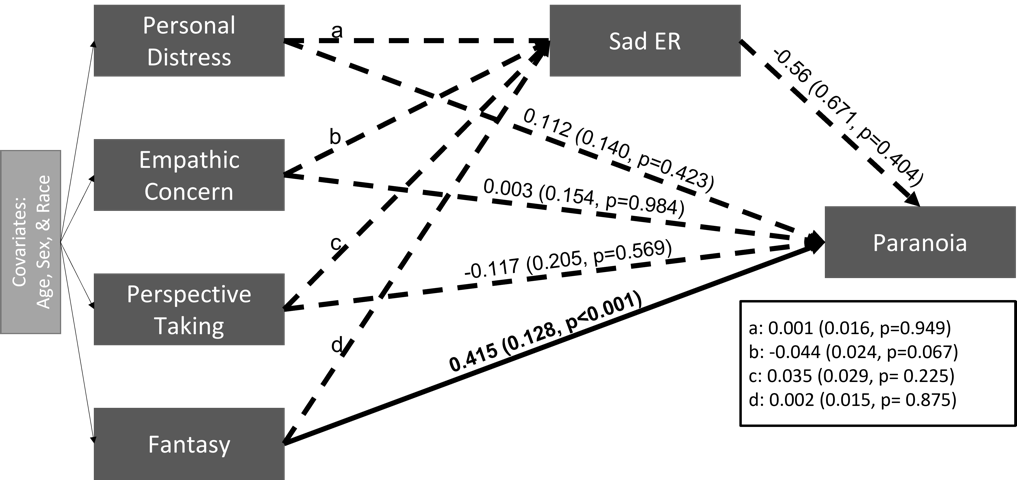


The estimate and standard error are presented for each path. Significant paths are indicated with solid black lines and bolded coefficients. Non-significant pathways are indicated with dashed lines.

**Figure S4: Path model with accuracy for anger emotion recognition**


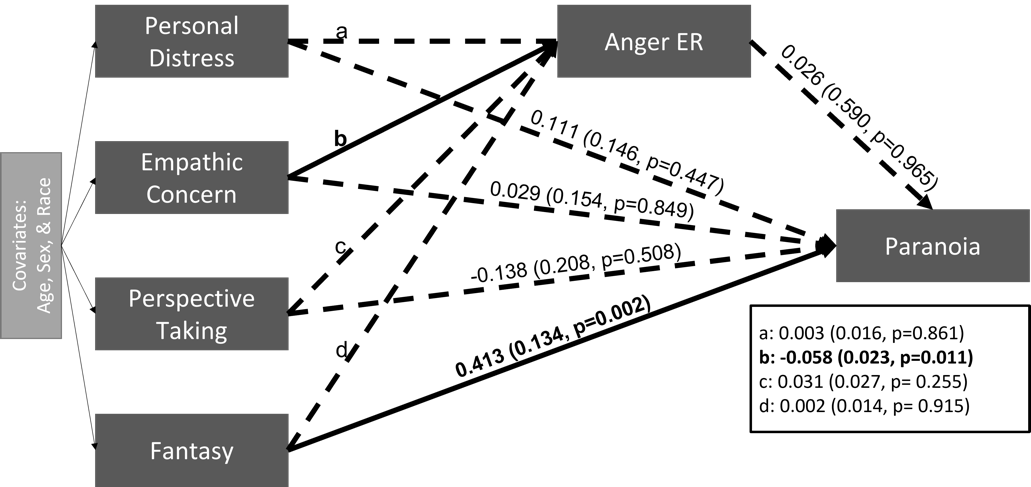


The estimate and standard error are presented for each path. Significant paths are indicated with solid black lines and bolded coefficients. Non-significant pathways are indicated with dashed lines.
